# A Noise Control Method Using Adaptive Adjustable Parametric Array Loudspeaker to Eliminate Environmental Noise in Real Time

**DOI:** 10.3390/ijerph19010269

**Published:** 2021-12-27

**Authors:** Yinsheng Li, Wei Zheng

**Affiliations:** Key Laboratory for Optoelectronic Technology and System of the Education Ministry of China, College of Optoelectronic Engineering, Chongqing University, Chongqing 400044, China; yinsheng.li@cqu.edu.cn

**Keywords:** ultrasound, environmental noise, active noise control, adjustable PAL, quiet areas

## Abstract

Long-term exposure to environmental noise is dangerous to human health. Therefore, there is an urgent need to suppress or eliminate environmental noise. Due to the limitation of environmental space, the use of reverse sound waves emitted by loudspeakers for noise elimination has been widely used in noise control. However, because of the omni-directionality of sound propagation, a traditional voice coil loudspeaker (VCL) is used as a secondary source (emission reverse sound wave). It is easy to increase the sound pressure in non-target areas and form significant acoustic feedback to the reference source. Therefore, we propose an online secondary path modeling method using an adjustable parametric array loudspeaker (PAL) based on ultrasounds to eliminate environmental noise in real time. According to the different distance of the target, the size of the PAL is adjusted adaptively to realize the noise control of different long-distance targets. The distribution of quiet areas is discussed. The experimental results showed that a PAL as a secondary source had the same noise reduction effect as a traditional VCL, but it had longer propagation distance, smaller sound feedback and a more regular and controllable distribution of quiet areas. These research findings have great potential for improving environmental noise and creating a quiet environment.

## 1. Introduction

Environmental noise is everywhere. However, when people work and live in a high-noise environment for a long time, it brings great risks to people’s health, such as hearing loss and noise trouble [1,2]. As people mainly work and live in indoor environments, the application scenarios of noise control are also mainly aimed at indoor environmental noise. At present, environmental noise control methods such as sound absorption and vibration isolation are mainly used. These methods have a good effect on reducing medium and high frequency noise in space. However, the passive noise control equipment is complex, and the low-frequency control effect is poor, which is greatly restrictive. In recent years, active noise control (ANC) can effectively make up for the deficiencies of passive noise control, and has become a hot research topic in the field of noise control [3,4]. With the development of adaptive active control technology, active noise control has been widely used in offices, automobiles and other spaces [5,6].

Active noise control, through the adaptive filtering algorithm, controls the secondary source to produce a secondary sound field, which is superimposed with the primary sound field, so as to achieve the purpose of noise suppression [7]. Adaptive filtering algorithm is the focus of active noise control. The well-known filtered-x least mean square (FxLMS) algorithm was proposed by Burgess in 1981 [8]. The FxLMS algorithm is still the most widely used because of its simplicity and efficiency. The FxLMS algorithm uses the secondary path response function to filter the reference signal, so the secondary path needs to be fitted. The secondary path fitting methods include online fitting and offline fitting. In practical use, the secondary path response function is changed due to the change of environment and device aging, so it is necessary to estimate the secondary path online. Eriksson et al., used the method of injecting auxiliary noise to fit the secondary path online for the first time [9]. In recent years, many researchers have used different strategies to improve the convergence speed and steady-state performance of the online secondary path modeling [10,11,12,13].

Active noise control uses traditional voice coil loudspeakers (VCLs) as the secondary source to suppress the noise in the target areas. Due to the omni-directivity of sound propagation, noise reduction is achieved at the target point, and the overflow of reverse sound waves increases the sound pressure level (SPL) of other adjacent areas [14,15]. When a multi-channel ANC system is used, the noise acquisition of the error microphone between the secondary sources produces crosstalk, which increases the additional calculation cost in the control process [16,17]. Due to the omni-directivity of sound propagation in traditional voice coil loudspeakers, it is easy to increase the acoustic feedback to the reference signal [18,19]. Therefore, there are still many shortcomings to using a traditional voice coil loudspeaker as a secondary source for noise control.

Ultrasound is a sound wave with a frequency higher than 20 kHz. It is used in parametric array loudspeakers by using the directivity of ultrasound. Parametric array loudspeakers (PALs) use the nonlinear effect of ultrasound wave in the medium to produce audible sound. Compared with traditional voice coil loudspeakers, parametric array loudspeakers, at both low frequency and high frequency, have significant high directivity [20,21]. Therefore, PALs can produce audible sound in the target areas without interfering with other areas [22]. In active noise control, researchers have used a PAL as the secondary source, which verified its feasibility [23,24,25]. More and more successful attempts have been made to use a PAL as a secondary source. Ganguly et al., used one as the secondary source to control the noise of the 1.06 m target [26]. Tanaka et al., used PALs to establish a silent area for the left and right ear areas of workers at 1.5 m [27]. However, in the above applications, the distance between the secondary source PAL and the noise reduction target is fixed and placed at a short distance, so it is impossible to adjust the distance of different targets adaptively. In addition, compared with traditional voice coil loudspeakers, the research on the many unique characteristics of PALs is still limited, and further research is still needed.

In previous studies using a PAL as a secondary source, its size and power were fixed. Because the spatial distance between the secondary source and the noise reduction target is fixed, once the target position is changed, new problems arise. When the target is too close to the secondary source, this can cause sound field overflow and multiple harmonics to form superposition and interference with the other adjacent sound fields. When it is too far, the noise reduction performance is greatly reduced. In view of the above research status, this paper uses the adjustable parametric array loudspeaker as the secondary source. According to the target distance, we adaptively adjusted the size and power of a PAL to achieve noise control in the target area. In addition, the size of acoustic feedback and the distribution of quiet areas between PALs and traditional VCLs are discussed and compared. Combined with the characteristics of PALs, it is helpful to improve the performance of ANC systems.

The rest of this paper is organized as follows. Section 2 presents the active noise control system based on online secondary path modeling and the theory of differential frequency sound field. In Section 3, experiments are carried out based on adjustable PALs and compared with traditional VCLs, and the experimental results are given. The conclusion is given in Section 4.

## 2. Methods

### 2.1. Online Secondary Path Modeling

Due to its simplicity and effectiveness, FxLMS algorithm is the most widely used adaptive algorithm. In the adaptive FxLMS algorithm, we need to identify the secondary path transfer function to obtain its estimated value. The process of using adaptive filtering principle to estimate the secondary path is called secondary path modeling. Secondary path modeling is divided into offline estimation and online estimation. The offline estimation is applicable to the application scenarios where the secondary path is stable or changes slowly. In this paper, the secondary path between the secondary source and the error microphone changes with the noise reduction target position, and the corresponding secondary path response function S(z) also changes. Therefore, the secondary path can only be used for online estimation.

Online secondary path modeling is employed to estimate the transfer function of the secondary path in real time according to the changes of the actual environment in the process of active noise control. The power of auxiliary noise affects the convergence speed of the secondary path fitting filter. The larger the auxiliary noise power, the faster the convergence speed of the secondary path filter, but it decreases the noise reduction performance of the system. When the auxiliary noise is too low, the convergence speed is reduced, which affects the stability of the ANC filter [11,12]. In order to balance the contradiction between the two, researchers proposed a method of variable power auxiliary noise. In the initial stage of system operation or when the secondary path changes, high-power auxiliary noise is used to speed up the convergence speed. When the system is stable, low power auxiliary noise is used. The most widely used is the auxiliary noise power regulation strategy proposed by Akhtar and Carini [11,28]. On this basis, this article adopts a more sensitive auxiliary noise power adjustment method.

**Figure 1:**
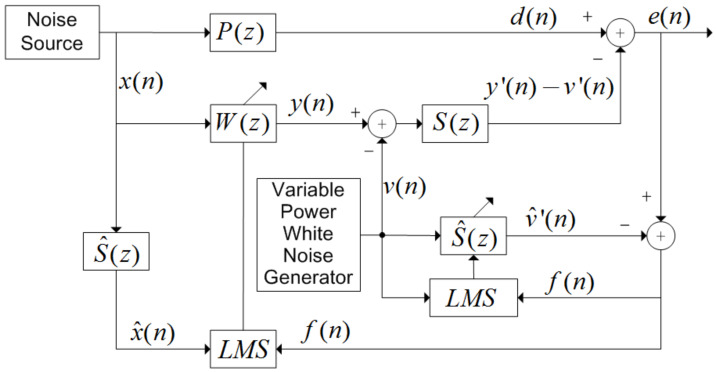
Online secondary path modeling with variable power auxiliary noise. x(n) is the signal of noise source, P(z) is the primary path transfer function, and the weight vector W(z) is iteratively updated by LMS algorithm.

Figure 1 shows the online secondary path modeling with variable power auxiliary noise. Among them, S(z) is the transfer function of the secondary path, and $\hat{S}(z)$ is the estimated quantity of the transfer function of the secondary path. The white noise generator generates a white noise signal v(n) that is not related to the reference signal x(n), and superimposes it with the output signal y(n) generated by the ANC filter. After passing through the secondary path S(z), y′(n)−v′(n) is obtained, and superimposed with the desired signal d(n), the error signal of the entire ANC system is

(1) $$e(n)=d(n)-y^{\prime }(n)+\upsilon^{\prime }(n).$$

After the white noise signal v(n) passes through the secondary pass filter, the estimated quantity $\hat{v}\prime (n)$ is obtained, and after superimposing it with the error signal e(n), the error quantity f(n) used to update the ANC filter coefficient and the secondary path filter coefficient is obtained:

(2) $$\begin{array}{l} f(n)=e(n)-\hat{v}\prime (n)\\ \;\;\;\;\;\;\;=d(n)-y\prime (n)+v\prime (n)-\hat{v}\prime (n)\\ \;\;\;\;\;\;\;\;=e_{x}(n)+e_{\upsilon }(n) \end{array}$$

Among them, $e_{x}(n)=d(n)-y\prime (n)$, $e_{\upsilon }(n)=v\prime (n)-\hat{v}\prime (n)$. $e_{x}(n)$ represents the error caused by the ANC filter, and $e_{\upsilon }(n)$ represents the error caused by the secondary path filter. The ANC filter and secondary path filter coefficient update formula is:

(3) $$\hat{S}(n+1)=\hat{S}(n)+\mu_{s}v(n)f(n),$$

(4) $$W(n+1)=W(n)+\mu_{w}\hat{x}\prime (n)f(n).$$

Among them, $\mu_{s}$ and $\mu_{w}$ are the step factors of the secondary path filter and the ANC filter, respectively. The white noise signal input vector is denoted as $v^{Τ}(n)={\left[v(n),v(n-1),\cdots ,v(n-L+1)\right]}^{Τ}$.

The commonly used auxiliary noise v(n) power adjustment method is:

(5) $$v_{g}(n)=\sqrt{(1-\rho (n))\sigma_{v_{\min}}^{2}+\rho (n)\sigma_{v_{\max}}^{2}}\;\;\;\;•\;\;v(n)=G(n)v(n),$$

(6) $$\rho (n)=\frac{p_{f}(n)}{p_{e}(n)}=\frac{p_{\left[d(n)-y^{\prime }(n)\right]}+p_{\left[v^{\prime }(n)-{\hat{v}}^{\prime }(n)\right]}}{p_{\left[d(n)-y^{\prime }(n)\right]}+p_{\left[v^{\prime }(n)\right]}},$$

$p_{f}(n)$ and $p_{e}(n)$ are the power of the error signal f(n) and e(n) respectively. G(n) is the auxiliary noise gain, $v_{g}(n)$ is the adjusted auxiliary noise signal, $\sigma_{v_{\min}}^{2}$ and $\sigma_{v_{\max}}^{2}$ are the minimum and maximum noise power, respectively. When the reference noise power is too large, the power that needs to be controlled at the error microphone is much greater than the auxiliary noise power, which causes large fluctuations in the secondary path filter. Therefore, it is necessary to consider the influence of the reference noise power when performing auxiliary noise power control.

For this reason, the ratio of the residual error power of the ANC filter to the power of the auxiliary noise filtered by the secondary path can be a fixed value.

(7) $$\frac{E({\left(d(n)-y^{\prime }(n)\right)}^{2})}{E({\left({v^{\prime }}_{g}(n)\right)}^{2})}=R=constant.$$

When the gain G(n) of the auxiliary noise changes slowly,

(8) $$E({\left({v^{\prime }}_{g}(n)\right)}^{2})=G^{2}(n)||s(n)|{|}^{2}E(v{\left(n\right)}^{2}),$$

||s(n)|| is the Euclidean norm of the secondary path coefficient vector.

(9) $$E(e{\left(n\right)}^{2})=E({\left(d(n)-y^{\prime }(n)\right)}^{2})+E({\left({v^{\prime }}_{g}(n)\right)}^{2}).$$

According to Equations (7)–(9), the calculation formula of the auxiliary noise gain can be obtained as:

(10) $$G(n)=\sqrt{\frac{P_{e}(n)}{\left(R+1)P_{\hat{s}}(n\right)}}.$$

Among them, $P_{\hat{s}}(n)$ also uses exponential smoothing to estimate:

(11) $$P_{\hat{s}}(n)=\lambda P_{\hat{s}}(n-1)+(1-\lambda ){\hat{s}}^{T}(n)\hat{s}(n),$$

λ is a forgetting factor close to 1. Because the residual noise is also related to the power level of the reference noise to a certain extent, the power of the residual noise may not fully reflect the closeness of the ANC filter to the steady state. In order to make the auxiliary noise power more sensitive to the state of ANC filter, the ratio of $P_{x}(n)$ and $P_{e}(n)$ is used to estimate the degree of convergence of the system.

(12) $$R(n)=\frac{P_{x}(n)}{P_{e}(n)}.$$

Compared with the fixed scale coefficient, the auxiliary noise power has smaller power in the steady state. The calculation formula of power gain is:

(13) $$G(n)=\sqrt{\frac{P_{e}(n)}{\left(R(n)+1)P_{\hat{s}}(n\right)}}.$$

Therefore, the auxiliary noise power adjustment formula is:

(14) $$v_{g}(n)=G(n)v(n)=\sqrt{\frac{P_{e}(n)}{\left(R(n)+1)P_{\hat{s}}(n\right)}}\;\;\;\;•\;\;\;v(n).$$

The above power regulation method is more sensitive to the state of ANC filter, has large power in the initial stage of system operation, can attenuate rapidly when the system tends to be stable, and the auxiliary noise power is greatly reduced in the steady state.

**Figure 2:**
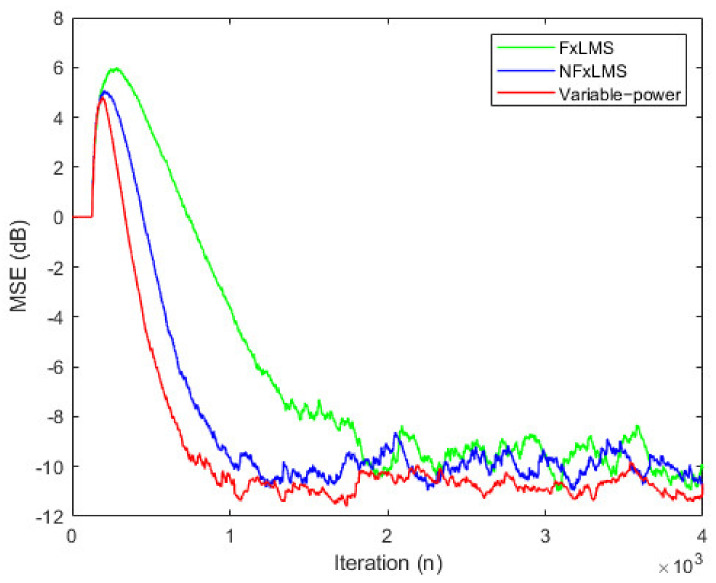
Performance comparison. The horizontal axis is iteration (n) and the vertical axis is MSE (dB). Legend is FxLMS, NFxLMS, and variable-power, respectively.

The existing FxLMS algorithm, normalized FxLMS algorithm and the online modeling method with variable power auxiliary noise used in this paper are simulated, respectively. The iterative mean square error (MSE) results are shown in Figure 2. The filter order of secondary path modeling is 32, and the iteration step of secondary path modeling is 0.01. It can be seen from the figure that the online modeling method with power auxiliary noise has faster convergence speed and lower steady-state offset.

### 2.2. Theoretical Basis of Parametric Array

Parametric array loudspeaker is a kind of parametric loudspeaker which can control the audible sound in a specific direction. Using the nonlinear effect of ultrasound wave in air, an audible sound field with high directivity is formed in air. The key of parametric array theory model is the establishment of nonlinear wave equation when sound wave propagates in air. Khokhlov, Zabolotskaya and Kuznetsov fully considered the absorption, scattering and nonlinear effects of finite amplitude sound beam in fluid and solid, and deduced the nonlinear equation of wave propagation in medium, which is the famous KZK equation [29,30]. The equation accurately describes the nonlinear propagation effect of sound wave in medium.

The expression of KZK equation is:

(15) $$\frac{\partial^{2}p}{\partial z\partial \tau }=\frac{c_{0}}{2}\nabla_{⊥}^{2}p+\frac{\delta }{2c_{0}^{3}}\frac{\partial^{3}p}{\partial \tau^{3}}+\frac{\beta }{2\rho_{0}c_{0}^{3}}\frac{\partial^{2}p^{2}}{\partial \tau^{2}},$$

where, p is the sound pressure, z is the propagation distance along the sound beam axis, $c_{0}$ is the sound velocity, $\tau =t-z/c_{0}$ is the delay time, δ is the sound scattering degree, β is the nonlinear coefficient, $\rho_{0}$ is the air density, and $\nabla_{⊥}^{2}$ is the XY plane Laplace operator perpendicular to the Z axis.

Because KZK equation involves many nonlinear parameters, such as scattering in the medium, heat propagation loss and molecular relaxation loss, it is difficult to obtain its exact analytical solution. The approximate solution or numerical solution is usually obtained by quasilinear method. It is assumed that the harmonic frequency component generated by the sound wave in the process of propagation has the fundamental frequency. Ignoring the third harmonic with small harmonic amplitude, it is only approximate to the second harmonic. At this time, the solution of KZK equation is:

(16) $$p=p_{1}+p_{2},$$

where, $p_{1}$ is the primary wave sound pressure generated by sound wave, and $p_{2}$ is the second harmonic generated by nonlinear propagation, and its amplitude is less than $p_{1}$.

Introducing complex pressure amplitude $q_{n}$ and set:

(17) $$p_{n}(r,z,\tau )=\frac{1}{2j}q_{n}(r,z)e^{jn\omega \tau }+c.c.\;\;\;\;\;\;\;\;\;\;\;\;\;\;\;\;\;\;n=1,2,$$

where, c.c. is the complex conjugate of the former term, and r is the distance between the projection of the midpoint of the sound field on the plane of the parametric array and the center of the plane. By applying Green’s function and Hankel’s transformation, the quasilinear solution of KZK equation is finally obtained as follows [31]:

(18) $$q_{1}(r,z)=2\pi \int_{0}^{\infty }q_{1}(r\prime ,0)G_{1}(r,z\left|r\prime ,0\right.)r\prime dr\prime ,$$

(19) $$q_{2}(r,z)=\frac{\pi \beta k}{\rho_{0}c_{0}^{2}}\int_{0}^{z}\int_{0}^{\infty }q_{1}^{2}(r\prime ,z\prime )G_{2}(r,z\left|r\prime ,z\prime \right.)r\prime dr\prime dz\prime ,$$

where, $k=\omega /c_{0}$ is the wave number, $G_{n}(r,z\left|r\prime ,z\prime \right.)$ is the Green function, $q_{1}(r,z)$ is the complex value of linear sound pressure of primary wave, and $q_{2}(r,z)$ is the complex value of second harmonic sound pressure caused by nonlinear propagation effect of primary wave. There are multiple integrals in the above formula, which is still not easy to calculate. By applying Hankel’s transformation to the wave equation, Liauh et al., obtained the calculation formula of general circular piston source parameter array parameters ϕ [32]:

(20) $$\phi (r,z)=\frac{Uaj}{2\pi k}\int_{-\pi }^{\pi }\frac{e^{-jkz}-e^{-jk\sqrt{R^{2}+z^{2}}}}{a-re^{j\psi }}d\psi ,$$

Among them, the particle vibration potential energy of the circular parameter array is evenly distributed on the plane with radius a, the amplitude is U, and the introduced variables R and ψ meet $R^{2}=(a-re^{-j\psi })(a-re^{j\psi })$.

The sound pressure at the (r,z) point can be expressed as:

(21) $$p(r,z,t)=-j\rho_{0}\omega e^{j\psi t}\phi (r,z),$$

The formula rewrites the original quadratic integral into one integral, which greatly reduces the computational complexity. By calculating each point in (r,z), the sound field distribution of parametric array can be easily obtained.

## 3. Noise Control Using Adjustable PAL

In this section, we introduce the hardware and experiments employed, and use an adjustable PAL to adjust adaptively for different distances. Based on this, we compare the noise reduction distance, acoustic feedback and noise reduction performance between the adjustable PAL and the traditional VCL. In addition, we also discuss the distribution of quiet areas.

### 3.1. Sound Field Distribution of PAL

**Figure 3:**
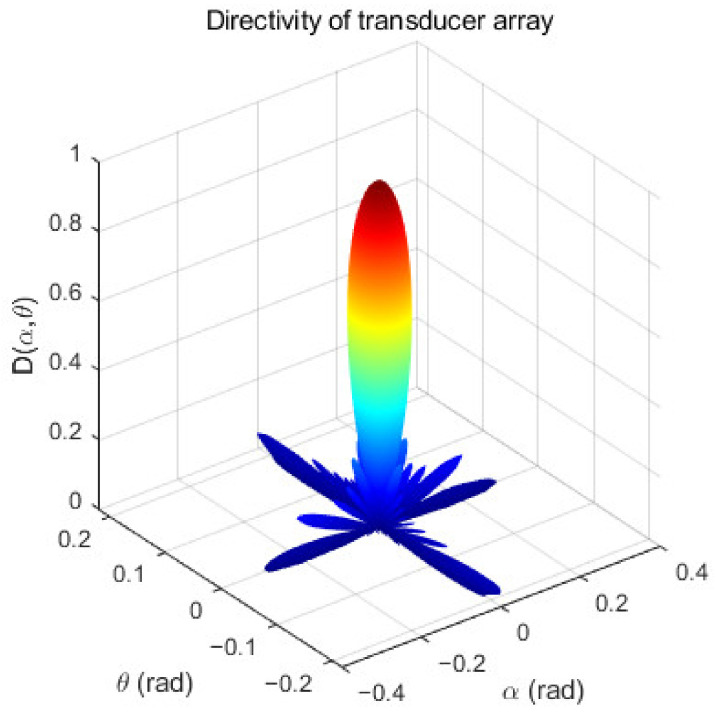
Transducer array directivity. The X-axis is angle α (rad), the Y-axis is angle θ (rad), and the Z-axis is directivity D(α,θ).

Parametric array loudspeakers produce high directivity audible sound by using the nonlinear propagation effect of ultrasound wave in the air [33]. The directivity of audible sound mainly depends on the directivity of ultrasound, and they are closely related. Formula 22 is the directivity formula of the ultrasound transducer. α is the angle between the projection of the directivity vector on the plane XOY of the transducer and the X axis, and θ is the angle between the directivity vector and the Z axis of the transducer, $J_{1}$ is the first-order Bessel functions, k = 2π/λ, λ = c/f, k is the wave number, λ is the wavelength, $\left(x_{i},y_{i}\right)$ is the position of the transducer, a is the radius of the transducer, and n is the number of the transducer. The units of the α,θ angle are rad; the unit of directivity is non-dimensional. The directivity of the transducer array is shown in Figure 3.

(22) $$D(\alpha ,\theta )=\left|\frac{2J_{1}(ka\sin\theta )}{ka\sin\theta }\right|\left|\sum_{i=1}^{n}e^{jk\left|x_{i}\sin\theta \sin\alpha +y_{i}\sin\theta \cos\alpha \right|}\right|,$$

The sound wave propagation of the traditional voice coil loudspeaker is omni-directional, and its direction is 360°. Assuming the emission direction of VCL as the axis, its semi directivity is 180°. The traditional voice coil loudspeakers do not easily produce high directivity audible sound, while the ultrasound transducer has good directivity, but the directivity of a single transducer is not high. The transducer array composed of multiple transducers has stronger directivity, and its side lobe is also effectively suppressed.

**Figure 4:**
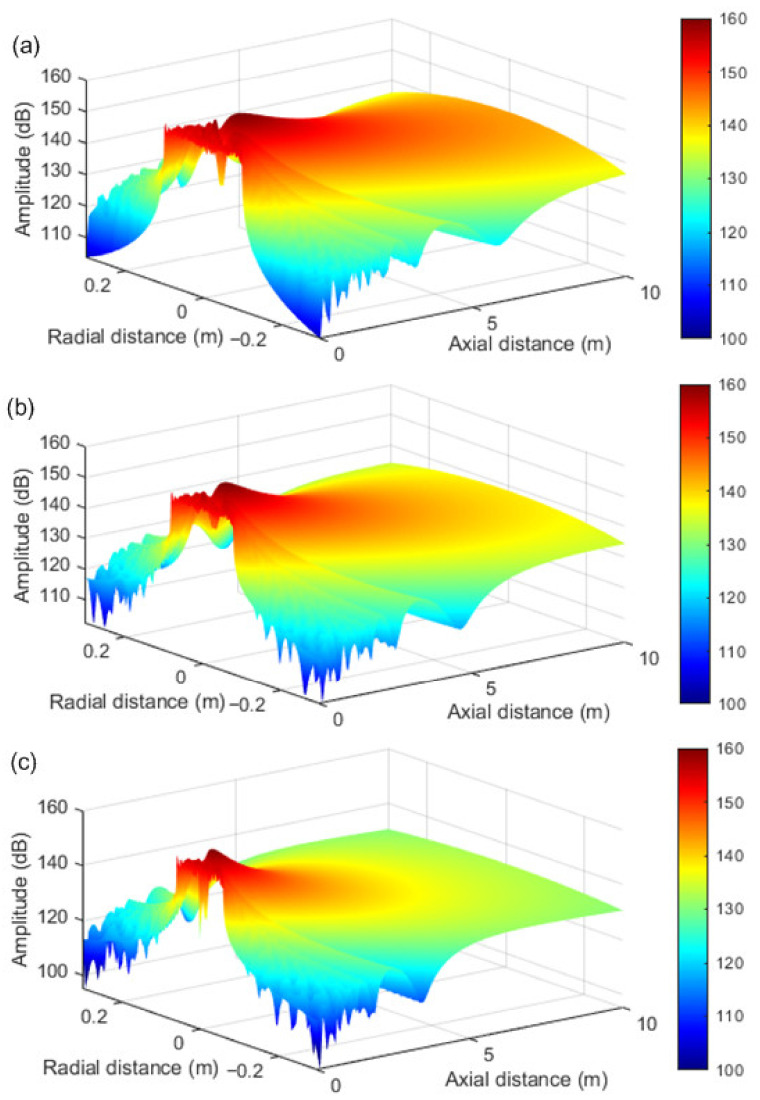
Sound field distribution of parametric array loudspeaker with different diameters. (**a**) 0.17 m in diameter; (**b**) 0.14 m in diameter; (**c**) 0.11 m in diameter.

The sound field generated by a PAL in air contains ultrasound signals, self-demodulating audible signals. In order to compare the sound field distribution and propagation distance of parametric arrays with different diameters, the composite sound field simulation was carried out according to the parametric array theory. This was performed under standard atmospheric pressure, at a temperature of 20 °C, a relative humidity of 30% RH, a carrier frequency of 40 KHz, and with the diameter of the parameter array at 0.17 m, 0.14 m and 0.11 m. The composite sound field distribution was shown in Figure 4; the unit of amplitude is dB. It can be seen from the figure that sound waves propagated approximately in bundles in the air, and the energy was mainly concentrated in the axial direction. The larger the diameter of the parametric array, the farther the propagation distance. It can be seen from the figure that the parametric array loudspeaker used in the control system had a maximum propagation distance of up to 10 m. The control system was used to eliminate the noise of close range (<10 m) targets. In addition, the smaller the diameter of the parametric array, the smaller the sound field coverage, and the smaller the interference to other adjacent sound fields.

### 3.2. Adaptive Adjustment of Noise Reduction Target Distance

**Figure 5:**
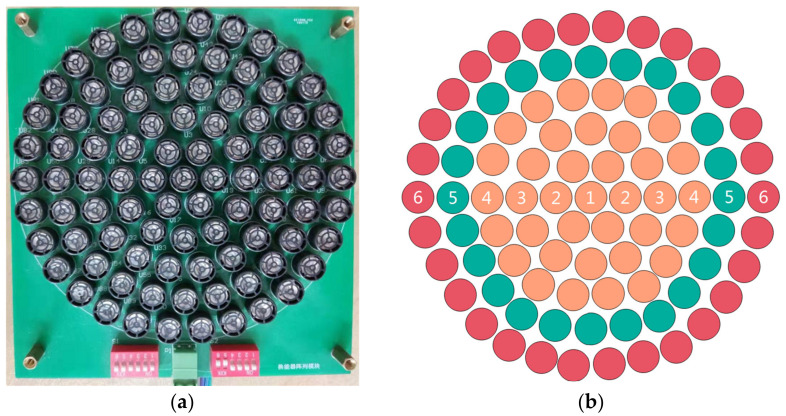
Adjustable PAL: (**a**) physical diagram; (**b**) schematic diagram.

The circular parametric array loudspeaker used in this paper is shown in Figure 5. The ultrasound frequency is 40 KHz, and the diameter of the parametric array loudspeaker is about 170 mm. The circular array consists of six layers; each layer can be freely controlled on and off. Due to the accumulation effect of parametric array ultrasound beam self-demodulation, the attenuation of audible sound in the propagation direction of the PAL is slower than the traditional VCL, and the propagation distance is longer. The acoustic propagation distance of the PAL is directly proportional to the amplitude of sound pressure and the size of parametric array. Therefore, this paper divided the PAL into three modes (M1 for 1–6 layers, M2 for 1–5 layers, M3 for 1–4 layers), as shown in Figure 5b. The number of ultrasound transducers corresponding to each mode of PAL is different. The larger the size, the farther the sound wave propagation distance, and the greater the corresponding power. We carried out experiments on them in turn.

**Figure 6:**
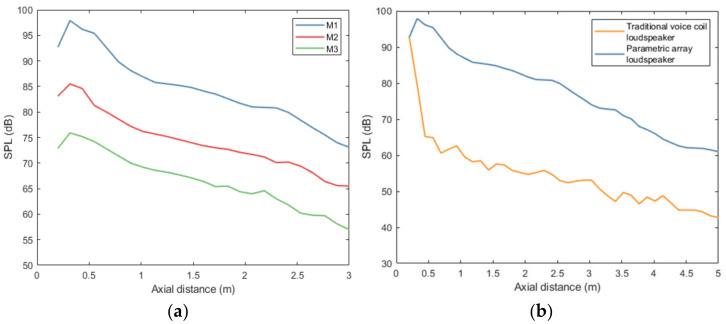
Axial sound pressure variation of traditional VCL and PAL: (**a**) axial sound pressure distribution of PAL in three modes; (**b**) axial sound pressure distribution of traditional VCL and PAL.

As the size of PAL increases, the corresponding propagation distance increases successively. The three modes of parametric array loudspeakers play white noise, and the axial sound pressure distribution is measured with a sound level meter, with an axial interval of 0.2 m. Its axial sound pressure distribution is shown in Figure 6a. Before the operation of the ANC system, the secondary source PAL sends ultrasound signal, and the error microphone synchronously detects the arrival time of the pulse, so as to calculate the distance between the error microphone and the secondary source, that is, the noise reduction target distance. According to the distance of the target, the size of the PAL can be freely controlled and the control distance can be roughly adjusted. If the target is far away, more layers are opened. If it is near, the outer layer is closed and only the inner layer is opened. When the same numbers of layers are opened, the fine adjustment of control distance can be realized by adjusting the power. Through the above strategy of coarse adjustment and fine adjustment, the noise reduction control distance of different targets can be adjusted adaptively. For short-range targets, due to the small sound field distribution range of the PAL with a smaller diameter, the use of a PAL with a smaller diameter can avoid the interference to the surrounding adjacent sound field.

In order to further compare the attenuation of sound pressure with distance, the PAL and traditional VCL played white noise. Their initial sound pressure was the same and their axial sound pressure distribution was measured, with an axial interval of 0.2 m. As shown in Figure 6b, with the increase in axial distance, the sound pressure attenuation of the traditional VCL was much larger than the PAL, and the average sound pressure difference between them was about 23.1 dB. Compared with the traditional VCL, the sound pressure of the PAL decreased slowly with the distance, and the propagation distance was longer. Using this characteristic, the PAL can achieve long-distance noise control.

### 3.3. Acoustic Feedback of Traditional VCL and PAL

**Figure 7:**
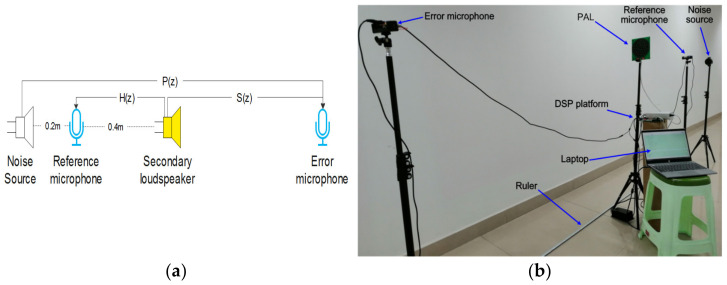
Experiment with laying scenes: (**a**) schematic diagram; (**b**) specific experimental scenarios.

Traditional VCLs and PALs were used as secondary source in turn to emit a white noise signal. The reference microphone and error microphone were placed as shown in Figure 7b. The distance between the secondary source and the error microphone was 3 m. All placement positions remained unchanged, and their corresponding signals were measured synchronously, corresponding to the feedback path H(z) and secondary path S(z) in Figure 7a. The DSP control platform with TMS320C6748 of TI Company as the core was adopted, and the signal sampling rate was 8 KHz. The feedback path and secondary path were identified by LMS algorithm. The number of taps of LMS filter was 128 and the update step was 0.001.

**Figure 8:**
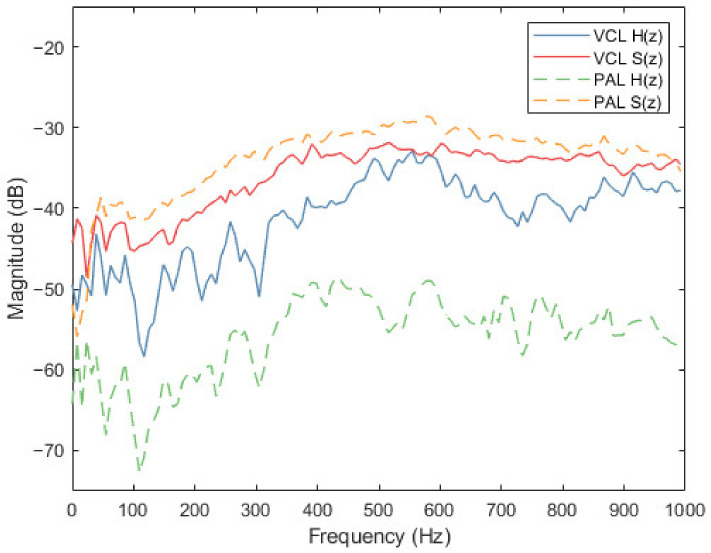
Amplitude frequency response of secondary path S(z) and feedback path H(z) of traditional VCL and PAL.

The amplitude frequency diagram of feedback path H(z) and secondary path S(z), corresponding to the traditional VCL and PAL, was shown in Figure 8. It can be seen from the figure that when the PAL was used as the secondary source, the amplitude of the feedback path was obviously smaller than the secondary path. The feedback path of the PAL was much smaller than that of the traditional VCL. As a secondary source, PALs can significantly reduce the sound feedback and make the system more stable.

### 3.4. Noise Reduction Performance of Traditional VCL and PAL

The experimental scenes are arranged according to Figure 7. Online secondary path modeling is carried out according to the noise reduction target. The DSP control platform with TMS320C6748 of TI Company as the core is adopted, and the signal sampling rate is 8 KHz. The step factor of ANC filter and secondary path filter is 0.001, and the tap length of the filter is 128. The noise source frequency is selected as 400 Hz, 600 Hz and 1000 Hz, respectively, and the narrowband multi-frequency noises are selected as 400 Hz + 600 Hz + 1000 Hz. In this paper, noise control is carried out in turn, and the distribution of noise reduction areas at a single frequency of 600 Hz was discussed.

**Figure 9:**
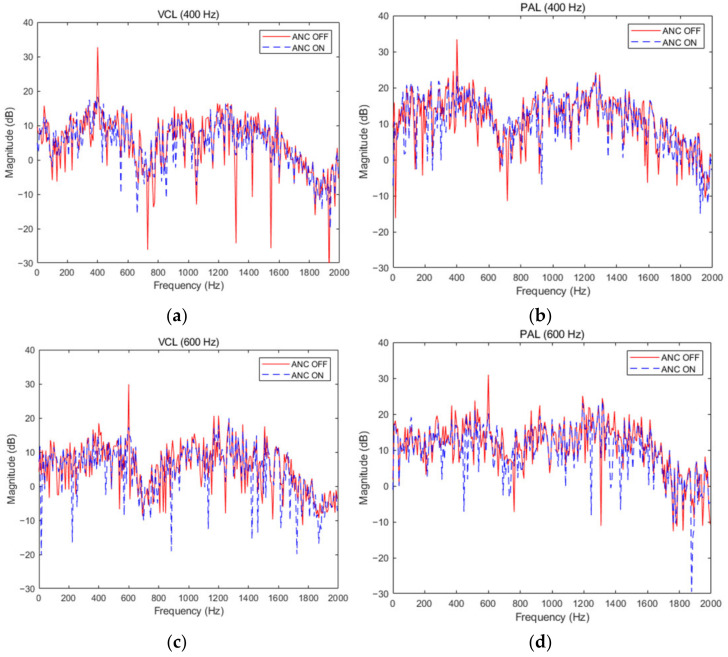
Noise reduction effect of traditional VCL and PAL at 400 Hz and 600 Hz. (**a**) VCL at 400 Hz; (**b**) PAL at 400 Hz; (**c**) VCL at 600 Hz; (**d**) PAL at 600 Hz.

**Figure 10:**
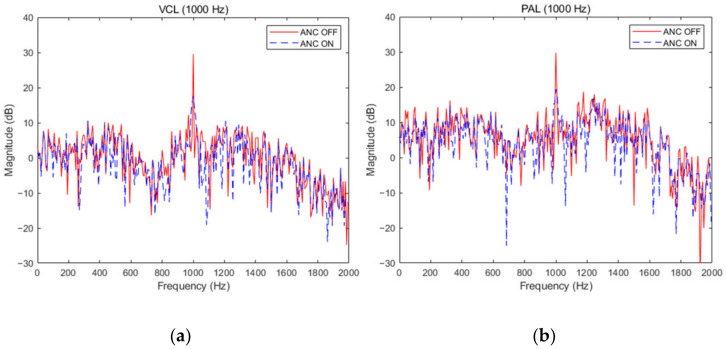

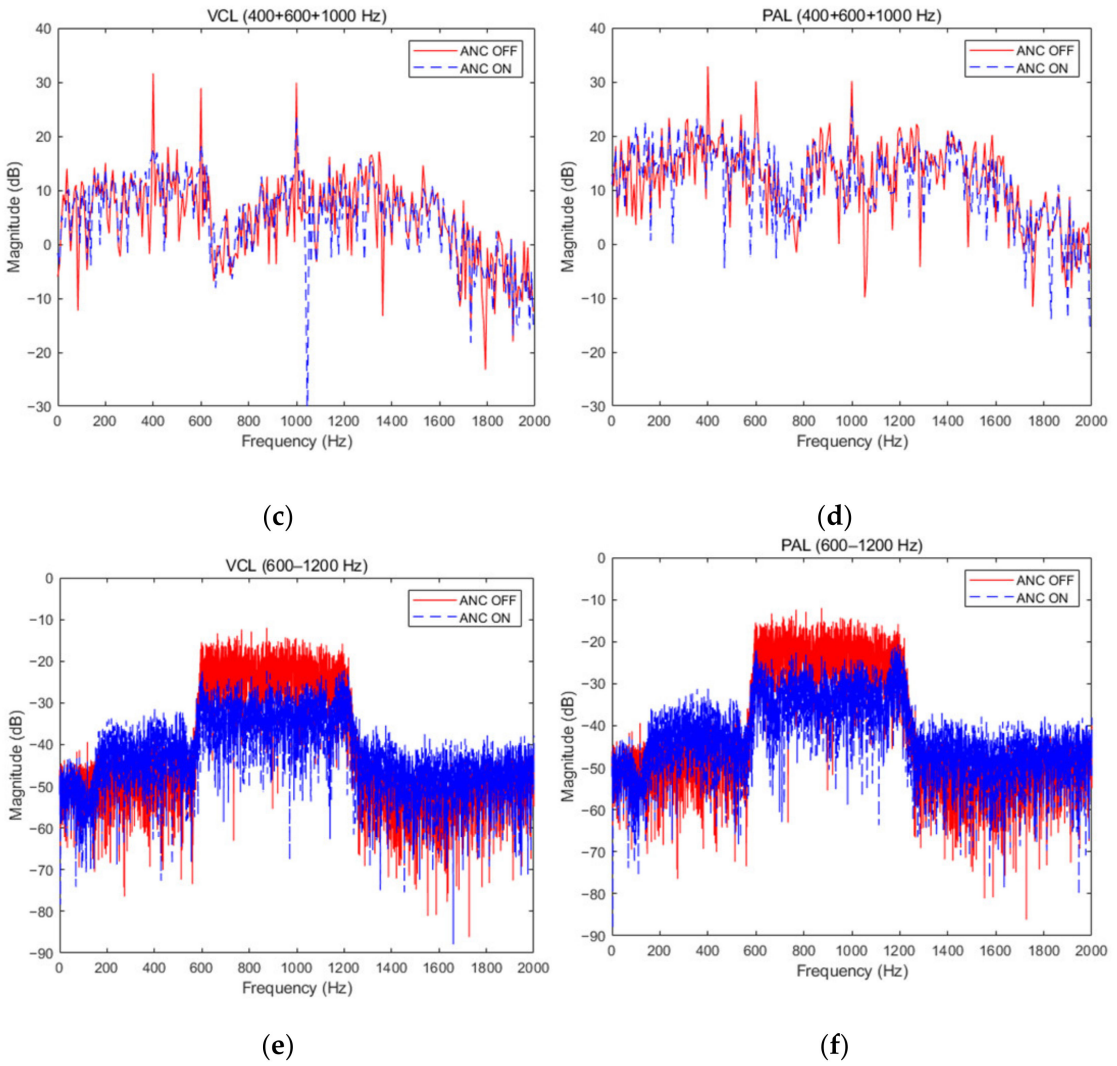
Noise reduction effect of traditional VCL and PAL at single frequency 1000 Hz, multi-frequency (400 Hz + 600 Hz + 1000 Hz) and narrow-band frequency (600 Hz–1200 Hz). (**a**) VCL at 1000 Hz; (**b**) PAL at 1000 Hz; (**c**) VCL at multi-frequency (400 Hz + 600 Hz + 1000 Hz); (**d**) PAL at multi-frequency (400 Hz + 600 Hz + 1000 Hz); (**e**) VCL at narrow-band frequency (600 Hz–1200 Hz); (**f**) PAL at narrow-band frequency (600 Hz–1200 Hz).

The noise reduction distribution of two kinds of secondary sources when the noise source is at the single frequencies of 400 Hz and 600 Hz was shown in Figure 9. When the single frequency was 400 Hz, the noise reduction of the PAL and traditional VCL was 15.1 dB and 14.4 dB. When the single frequency was 600 Hz, the noise reduction was 10.8 dB and 12.1 dB, respectively. When the noise source was at the single frequency of 1000 Hz and at multi-frequency, the noise reduction effect was shown in Figure 10. When the single frequency was 1000 Hz, the noise reduction was 11.4 dB and 12.0 dB.. The average noise reduction was 9.7 dB and 10.2 dB at multi-frequency. At single frequency and multi-frequency, the PAL and traditional VCL had the same active noise control effect. It could be seen from the two figures that the fundamental noise amplitude of PAL was higher than the traditional VCL. This was due to the accumulation effect of the PAL beam self-demodulation, which led to the reduction of fundamental frequency noise reduction. In addition, due to the influence of nonlinearity in the air, multiple harmonics will be generated in the signal propagation process, which will interfere with the effective signal and reduce the signal quality. When the noise is a narrow-band signal of 600 Hz–1200 Hz, there is no significant difference between the noise reduction effect of the parametric array loudspeaker and the traditional voice coil loudspeaker. The average noise reduction of the two is 11.5 dB and 12.3 dB, respectively. Therefore, the parametric array loudspeaker and the traditional voice coil loudspeaker have the same noise reduction effect as the secondary source in the active noise control system.

### 3.5. The Distribution of Noise Reduction Area

**Figure 11:**
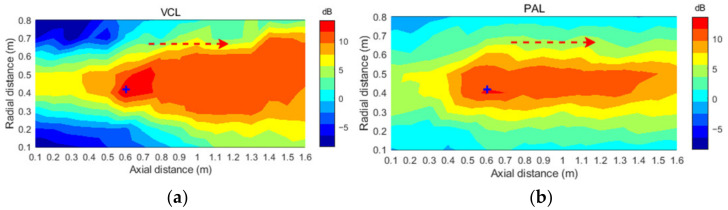
Noise reduction distribution of PAL and traditional VCL. (**a**) VCL; (**b**) PAL.

In order to compare the noise reduction areas distribution of traditional VCL and PAL, the noise reduction areas of them were measured. The noise reduction experiments with the above noise source at 600 Hz were taken as the object. The noise reduction distribution around the error microphone was measured. As shown in Figure 11, in a 0.8 m × 1.6 m rectangular area perpendicular to the ground, the sound pressure was measured at equal intervals with a particle size of 10 cm. A total of 306 sound pressure values were measured before and after the noise reduction, so as to intuitively reflect the change trend of the rectangular noise area before and after noise reduction. The “+” in Figure 11 was the position of the error microphone, and the noise source, secondary source and error microphone were in a straight line, as shown in Figure 7.

As shown in Figure 11, taking the error microphone as the starting point, a certain range of quiet area was established along the sound propagation direction (as indicated by the dashed red arrow). The noise reduction area of the PAL as secondary source was smaller than that of the traditional VCL. The boundary distribution of noise reduction areas of PAL was more controllable and approximately rectangular, while the traditional VCL was approximately fan-shaped. The PAL had obvious advantages in the application of noise reduction in a specific range. In the adjacent sound pressure distribution, the increment of the traditional VCL to other areas was 9 dB, while the increment of the PAL to adjacent areas was less than 2 dB. Due to noise control, the noise in other adjacent areas increases, so that people are exposed to a high noise environment, which will bring risks to people’s hearing [34]. Compared with the omni-directionality of the traditional voice coil loudspeaker, the parametric array loudspeaker has high directivity. When it is used as the secondary source, the interference of the reverse sound wave emitted by it to other adjacent areas can be ignored, and it does not cause the increase of sound pressure level in other areas. The sound field of the PAL is mainly concentrated in the axial area, and the area of its quiet area is smaller than that of the VCL, but the noise reduction area is mainly concentrated in the axial area, which is more controllable. Therefore, the PAL had the advantages of controllable noise reduction areas and small interference to adjacent areas. However, the current technique also has limitations, such as the control system producing harmonic distortion and fundamental frequency noise. It can be further solved by optimizing carrier signal modulation methods, such as improving signal broadband and spectrum utilization. The control system needs to place a real microphone at the target point to collect residual noise. In the future, the virtual microphone technology can be used to replace the real microphone, which has simplified the control system.

## 4. Conclusions

In this paper, the adjustable PAL is used as the secondary source emitting reverse noise waves to eliminate environmental noise, and the size and power of the PAL are adjusted adaptively according to the different noise target, so as to realize the active noise control in the target area. The secondary path modeling with variable power auxiliary noise can realize adaptive noise reduction for different long-range targets and make the system converge faster and have a lower steady-state offset. For indoor environments, the sound pressure attenuation of the traditional VCL was much greater than the PAL’s, which limited noise control of the traditional VCL to the short-range target only. The experimental results showed that by adjusting the power and radius of the PAL, the noise control of different distance targets could be realized. The PAL had the same noise reduction performance as the traditional VCL, and the maximum noise reduction was 15.1 dB. In the noise reduction areas distribution, although the PAL was smaller than the traditional VCL, the noise interference to other adjacent areas could be ignored, and the noise reduction areas were more controllable. These results have some practical guiding significance for environmental noise cancellation at long distance and specific range in indoor environments.

## Data Availability

The datasets used and/or analyzed during the current study are available from the corresponding author on reasonable request.
